# Managing Myotonic Dystrophy Type 1 Complicated by Metabolic Syndrome

**DOI:** 10.1016/j.jaccas.2024.102761

**Published:** 2024-12-04

**Authors:** Gianluca Pagnoni, Ashraf Nassar, Francesca Grossule, Matteo Paolini, Arianna Maini, Anna Vittoria Mattioli, Giuseppe Boriani, Francesca Coppi

**Affiliations:** a Cardiology Unit, Policlinico di Modena Hospital, Modena, Italy; b Technical and Applied Medical Sciences Sector at Alma Mater Studiorum, University of Bologna, Bologna, Italy; c Cardiology Division, Department of Biomedical, Metabolic and Neural Sciences, University of Modena and Reggio Emilia, Policlinico di Modena, Modena, Italy

**Keywords:** diabetes, dyslipidemias, exercise, genetics, hypercholesterolemia, lipid metabolism disorders, metabolic syndrome X

## Abstract

Myotonic dystrophy type 1 (MD1) is the most common form of muscular dystrophy in adults. MD1 is caused by the expansion of CTG repeats in the DMPK gene and affects various organs beyond muscles. We present a case of a patient with MD1 exhibiting features of metabolic syndrome (MetS), including insulin resistance and dyslipidemia. The patient was treated with PCSK9 inhibitors, ezetimibe, and bempedoic acid because of intolerance. Metabolic syndrome is more prevalent in patients with muscle disorders like MD1, primarily caused by the sedentary lifestyle associated with muscle weakness. Although no specific studies on MetS frequency in MD1 exist, data on its components are available. This case highlights the management of MetS in MD1 with innovative therapies. Managing metabolic syndrome in MD1 patients requires personalized therapies. This case introduces a promising therapeutic approach for statin-intolerant patients.

## Visual Summary

**Figure:**
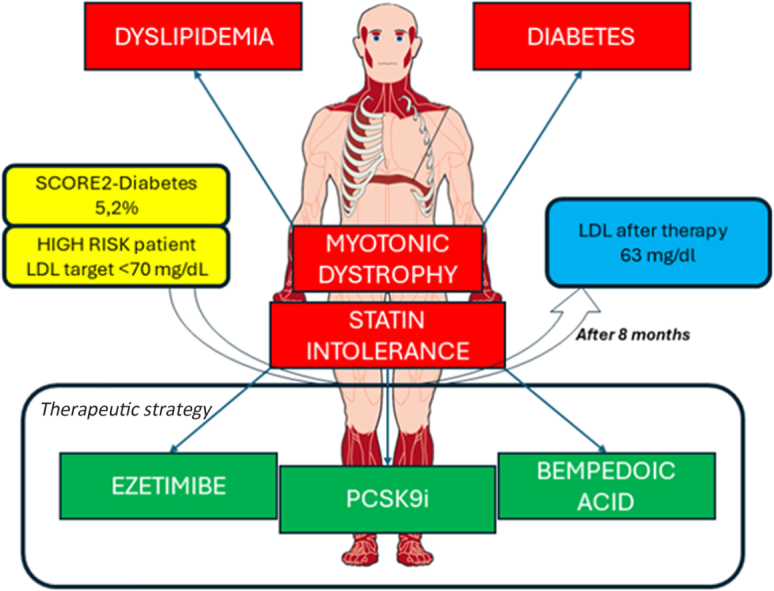

We present the case of a young female patient with type 1 myotonic dystrophy, concomitant nonalcoholic steatohepatitis, and mixed hyperlipidemia consistent with a metabolic syndrome phenotype. Due to intolerance to statin therapy, we opted for combination therapy with bempedoic acid and ezetimibe added to the proprotein convertase subtilisin/kexin type 9 (PCSK9) inhibitor. Literature indicates that bempedoic acid, when added to background PCSK9 inhibitor therapy, significantly lowers low-density lipoprotein cholesterol (LDL-C) by 30.3%.^1^

**Take-Home Messages**

• The combination of ezetimibe, a PCSK9 inhibitor, and bempedoic acid can be effective for patients with statin intolerance and high cardiovascular risk.
• Bempedoic acid offers a viable option for reducing LDL-C without significant muscle side effects.

## Initial presentation

The patient, a 52-year-old woman, was initially diagnosed with type 1 myotonic dystrophy through clinical manifestations and positive genetic testing. Clinical manifestations emerged around the age of 20 years, with progressive loss of strength in the upper limbs, bitemporal muscular atrophy, and bilateral palpebral ptosis along with persistently elevated serum creatine kinase levels above 400 IU/L. Genetic testing of the DMPK1 gene showed CTG triplet amplification consistent with the N/E1-E2 genotype of myotonic dystrophy type 1. At the time of reporting, a neurological evaluation reported progressively worsening manifestations, mainly consisting of mild bilateral palpebral ptosis, hyposthenia of distal upper and lower limbs, diffuse areflexia, and spontaneous and percussion myotonia, and the latest creatine kinase levels were 281 IU/L.

## Medical history

The patient was initially diagnosed with hyperlipidemia at the age of 44 years because of elevated total cholesterol, LDL-C, and triglyceride levels. She was then treated with ezetimibe monotherapy because of persistently elevated serum creatine kinase levels and relative contraindication and intolerance to statin therapy caused by known muscular disease. At follow-up visits at the local lipid clinic, elevated serum transaminase (aspartate aminotransferase/alanine aminotransferase) and gamma-glutamyl transferase levels prompted further liver diagnostic studies. Steatosis, consistent with the metabolic syndrome phenotype, was reported on abdominal ultrasound imaging.^2^

## Differential Diagnosis

The patient exhibited resistance to statin therapy caused by known muscular side effects, necessitating the exploration of alternative therapeutic options for managing her mixed dyslipidemia and conditions related to metabolic syndrome.

## Investigations

We performed cardiovascular risk calculation using the SCORE2-diabetes system for a moderate-risk country, resulting in a 10-year cardiovascular risk of 5.2%. Due to the overall comorbidity profile, we decided to switch the patient to PCSK9 inhibitors. After 1 month of therapy with evolocumab, total cholesterol was 307 mg/dL, high-density lipoprotein cholesterol was 80 mg/dL, LDL-C was 161 mg/dL, and triglycerides were 329 mg/dL. This modest result was likely not caused by poor adherence to therapy, which the patient reported as excellent, but rather caused by the laboratory tests being conducted too soon.

## Management

Due to persistently elevated total and LDL-C levels after 1 month, and with additional therapeutic approaches at our disposal, we considered adding the recently introduced bempedoic acid because its selective action is expected to spare muscle cells from any detrimental effects. Among statin-intolerant patients, treatment with bempedoic acid has been associated with a lower risk of major adverse cardiovascular events.^3^

After careful risk and benefit discussion with the patient, we initiated therapy with bempedoic acid at 180 mg/d. Further blood testing after 1 month demonstrated a marked reduction in cholesterol levels, with no observed muscular symptoms or laboratory signs of toxicity.

## Results and Follow-up

The combination therapy of ezetimibe, a PCSK9 inhibitor, and bempedoic acid reduced the patient's 10-year cardiovascular disease risk from 5.2% to 3.5%, as calculated using the SCORE2-Diabetes system. This treatment also achieved the target LDL-C level for high-risk patients (LDL-C <70 mg/dL), with our patient reaching an LDL-C level of 63 mg/dL, all without causing side effects.

## Discussion

The introduction of monoclonal antibodies targeting PCSK9 has substantially improved the therapeutic ability to reduce LDL-C levels and achieve recommended target values. These drugs act by inhibiting PCSK9, a molecule normally involved in transporting low-density lipoprotein (LDL) receptors to lysosomes for degradation. Inhibiting PCSK9 promotes the recycling of LDL receptors on the surface of hepatocytes, thereby increasing their capacity for LDL clearance from the circulation.^4^ The 2019 guidelines from the European Society of Cardiology and the European Atherosclerosis Society (ESC/EAS) for the management of dyslipidemia have established more stringent therapeutic targets for lipid-lowering therapy. For patients at high cardiovascular risk, a reduction in LDL-C levels of ≥50% is recommended, along with a target level of <1.8 mmol/L (<70 mg/dL). Meanwhile, for patients at very high cardiovascular risk, a reduction in LDL-C levels of ≥50% is recommended, along with a target level of <1.4 mmol/L (<55 mg/dL). To achieve these targets, the ESC/EAS 2019 guidelines recommend intensifying lipid-lowering therapy through increased use of combination therapies.^5^

In our case, we calculated cardiovascular risk using the SCORE2-diabetes system for a moderate-risk country, resulting in a 10-year cardiovascular risk of 5.2%. This classified the patient as high risk, with a target LDL level of <70 mg/dL. Unfortunately, the patient could not tolerate statins, so we initially used ezetimibe, achieving a modest LDL level of 201 mg/dL (Table 1). Consequently, we introduced the PCSK9 inhibitor evolocumab, which resulted in an initial LDL reduction to 161 mg/dL after 1 month (Table 2). To further optimize lipid-lowering therapy, we added bempedoic acid 1 month later. The decision to delay the introduction of bempedoic acid was clinical; given the patient's statin intolerance and overall condition, a gradual introduction of the new therapy was preferred.

**Table 1:** Baseline Lipid Panel During Ezetimibe Monotherapy

Total Cholesterol, mg/dL	LDL Cholesterol, mg/dL	HDL Cholesterol, mg/dL	Triglycerides, mg/dL	Creatine kinase, IU/L
328	194	87	232	326

HDL = high-density lipoprotein; LDL = low-density lipoprotein.

**Table 2:** Lipid Panel After 1 Month of PCSK9-Inhibitor Therapy + Ezetimibe

Total Cholesterol, mg/dL	LDL Cholesterol, mg/dL	HDL Cholesterol, mg/dL	Triglycerides, mg/dL	Creatine kinase, IU/L
307	161	80	329	281

Abbreviations as in Table 1.

This is particularly evident in women, as in the case of the patient, where there is an increased risk of cardiovascular diseases among women with type 2 diabetes mellitus.^6^^,^^7^ Furthermore, stress and anxiety associated with syndromic diseases can lead to the development of an unhealthy lifestyle, which promotes the onset of obesity in women. Obesity is linked to inflammation and endothelial dysfunction, which are pathophysiological foundations for atherosclerosis.^6^, ^7^, ^8^, ^9^

**Figure 1:**
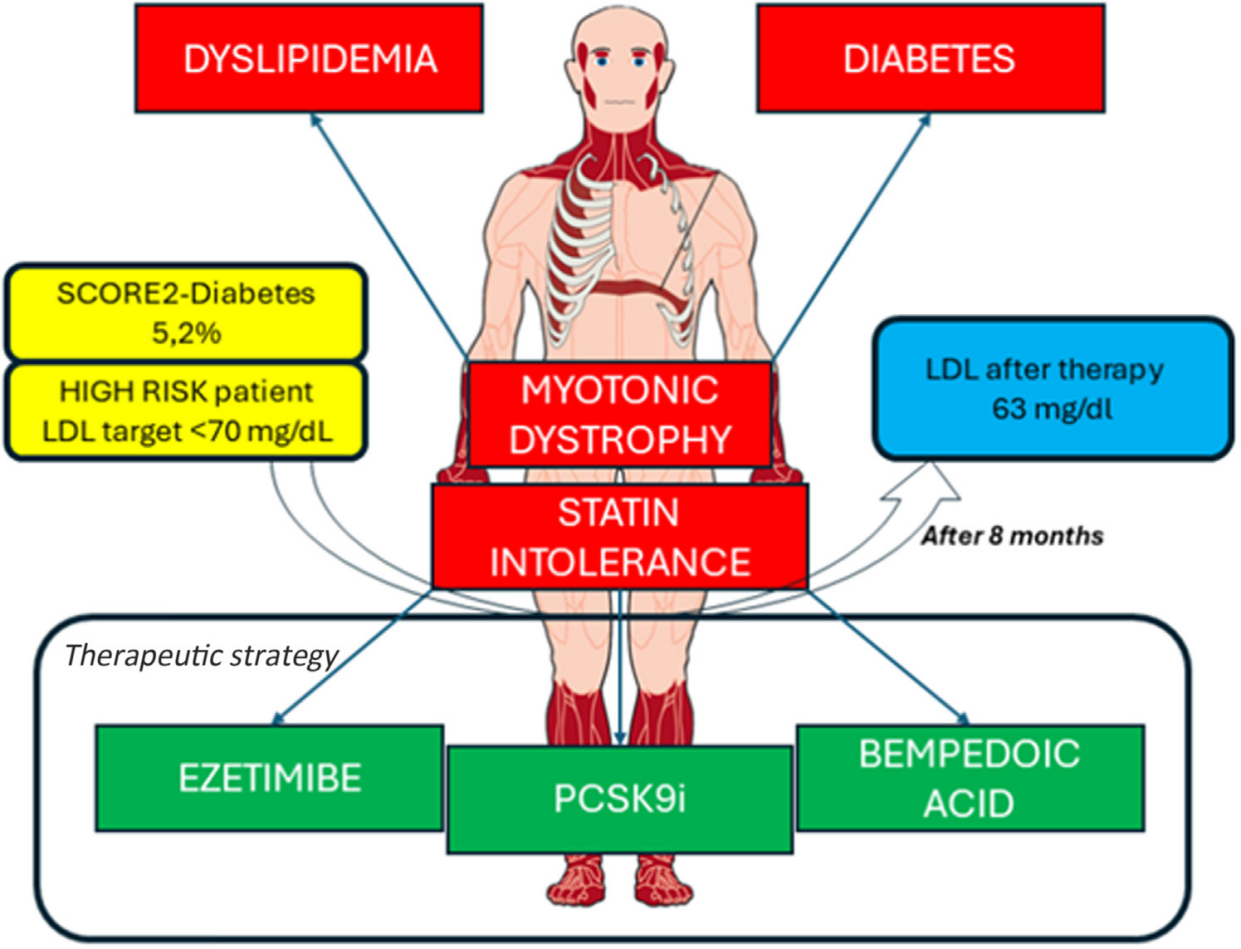
Patient's Conditions, Cardiovascular Risk, and Treatment Outcomes In the red boxes, the patient's medical conditions are listed: myotonic dystrophy, dyslipidemia, diabetes and statin intolerance. In the yellow boxes, the patient's cardiovascular risk is calculated using SCORE2-Diabetes, with an LDL target of <70 mg/dL. In the green boxes, the prescribed medical therapy is shown: ezetimibe + PCSK9i + bempedoic acid. In the blue box, the low-density lipoprotein (LDL) value achieved after 8 months of therapy is displayed.

Bempedoic acid was chosen for its action that spares muscle cells from detrimental effects. Bempedoic acid is an ATP citrate lyase inhibitor that targets cholesterol synthesis upstream of 3-hydroxy-3-methylglutaryl-coenzyme A reductase, the enzyme inhibited by statins. By reducing hepatic cholesterol synthesis and increasing LDL receptor expression, bempedoic acid enhances LDL clearance from circulation without activating in muscle tissue, thereby minimizing the risk of muscle-related side effects.^10^ The introduction of bempedoic acid to the combination therapy resulted in a significant reduction in LDL levels, reaching the target of <70 mg/dL without causing muscle side effects. As shown in Table 3, LDL dropped to 95 mg/dL after 1 month of combination therapy with bempedoic acid and ezetimibe, and further to 63 mg/dL after 8 months (Table 4, Figure 1).

**Table 3:** Lipid Panel After 2 Months of PCSK9-Inhibitor and 1 Month of Bempedoic Acid Initiation + Ezetimibe

Total Cholesterol, mg/dL	LDL Cholesterol, mg/dL	HDL Cholesterol, mg/dL	Triglycerides, mg/dL	Creatine kinase, IU/L
180	95	85	278	224

Abbreviations as in Table 1.

**Table 4:** Lipid Panel After 8 Months of PCSK9-Inhibitor and 6 Months of Bempedoic Acid Initiation + Ezetimibe

Total Cholesterol, mg/dL	LDL Cholesterol, mg/dL	HDL Cholesterol, mg/dL	Triglycerides, mg/dL	Creatine kinase, IU/L
100	63	54	189	337

Abbreviations as in Table 1.

This case demonstrates the effectiveness of combination therapy in achieving LDL-C targets in high-risk patients who are statin intolerant. Additionally, the use of bempedoic acid as part of the combination therapy offers a valid alternative for patients with statin intolerance, ensuring significant LDL-C reduction without the muscle-related side effects. It is important to consider a gradual approach when introducing new drugs to effectively manage complex conditions in patients with multiple comorbidities.

## Conclusions

This case report focuses on a patient subgroup not fully addressed by current guidelines. It details the successful treatment of a patient with myotonic dystrophy type 1, metabolic syndrome, and statin intolerance, who has a high 10-year cardiovascular risk of 5.2%. The patient was treated with a combination of ezetimibe, a PCSK9 inhibitor, and bempedoic acid, achieving the therapeutic goal of reducing LDL-C to below 70 mg/dL. Specifically, the patient's LDL was lowered to 63 mg/dL without any complications. This case underscores the need for longer-term evaluation and larger studies to understand the potential benefits of this treatment strategy for similar patients.

**Table:** VISUAL SUMMARY: Managing Myotonic Dystrophy Type 1 Complicated by Metabolic Syndrome

Timeline of Case Progression 1. Initial Presentation (Age 20 years) ○ Symptoms: Progressive loss of strength in upper limbs, bitemporal muscular atrophy, bilateral palpebral ptosis. ○ Tests: Elevated serum creatine kinase levels >400 IU/L, genetic testing confirmed CTG triplet amplification in DMPK1 gene. 2. Medical History (Age 44 years) ○ Diagnosis: Hyperlipidemia with elevated total cholesterol, LDL-C, and triglycerides. ○ Treatment: Ezetimibe monotherapy caused by intolerance to statin therapy. 3. Follow-up (Age 52 years) ○ Liver Function: Elevated aspartate aminotransferase/alanine aminotransferase and gamma-glutamyl transferase levels; abdominal ultrasound revealed steatosis. 4. Investigations ○ Cardiovascular Risk Calculation: 10-year risk of 5.2% using SCORE2-diabetes system. 5. Management Plan ○ Initial Treatment: PCSK9 inhibitor (evolocumab). ○ Results after 1 Month: • Total cholesterol: 307 mg/dL • LDL cholesterol: 161 mg/dL • high-density lipoprotein cholesterol: 80 mg/dL • Triglycerides: 329 mg/dL 6. Therapy Adjustment ○ Addition of Bempedoic Acid: Due to persistently elevated cholesterol levels and selective action sparing muscle cells. 7. Results After Adding Bempedoic Acid ○ 1 Month: Significant reduction in cholesterol levels. ○ 8 Months: Achieved target LDL levels (63 mg/dL) without muscle side effects. 8. Follow-Up and Outcomes ○ 10-Year Cardiovascular Risk Reduction: From 5.2% to 3.5%. ○ Achieved LDL Target: <70 mg/dL, no side effects.

## Funding Support and Author Disclosures

The authors have reported that they have no relationships relevant to the contents of this paper to disclose.
